# From data to discovery: Technology propels speech-language research and theory-building in developmental science

**DOI:** 10.1016/j.neubiorev.2025.106199

**Published:** 2025-05-05

**Authors:** Zuzanna Laudańska, Anna Caunt, Alejandrina Cristia, Anne Warlaumont, Katerina Patsis, Przemysław Tomalski, Petra Warreyn, Drew H. Abney, Jeremy I. Borjon, Manu Airaksinen, Emily JH Jones, Sven Bölte, Magdalena Dall, Daniel Holzinger, Luise Poustka, Herbert Roeyers, Sam Wass, Dajie Zhang, Peter B. Marschik

**Affiliations:** a Department of Child and Adolescent Psychiatry, Heidelberg University Hospital, Heidelberg University, and German Center for Mental Health (DZPG), Heidelberg, Germany; b Institute of Psychology, Polish Academy of Sciences, Warsaw, Poland; c School of Psychology, University of Plymouth, UK; d Laboratoire de Sciences Cognitives et de Psycholinguistique, Département d’études cognitives, ENS, EHESS, CNRS, PSL University, France; e Departments of Communication and Psychology, University of California, Los Angeles, USA; f Department of Experimental Clinical and Health Psychology, Ghent University, Belgium; g Department of Psychology, University of Georgia, Athens, USA; h Department of Psychology, University of Houston, Houston, USA; i Texas Institute for Measurement, Evaluation, and Statistics, University of Houston, Houston, USA; j Texas Center for Learning Disorders, University of Houston, Houston, USA; k BABA Center, Department of Physiology, University of Helsinki, Finland; l Department of Psychology, Birkbeck, University of London, and Centre for Developmental Neurobiology and Department of Child and Adolescent Psychiatry, IoPPN, King’s College London, United Kingdom; m Center of Neurodevelopmental Disorders (KIND), Department of Women’s and Children’s Health, Centre for Psychiatry Research, Karolinska Institutet & Region Stockholm, Stockholm, Sweden; n Child and Adolescent Psychiatry, Stockholm Health Care Services, Region Stockholm, Stockholm, Sweden; o Curtin Autism Research Group, Curtin School of Allied Health, Curtin University, Perth, Australia; p Research Institute for Developmental Medicine, Johannes Kepler University Linz, Linz, Austria; q Institute for the Science of Early Years, University of East London, London, UK; r iDN – interdisciplinary Developmental Neuroscience, Division of Phoniatrics, Medical University of Graz, Austria; s Child and Adolescent Psychiatry and Psychotherapy, University Medical Center Göttingen, Leibnz ScienceCampus Primate Cognition and German Center for Child and Adolescent Health (DZKJ), Göttingen, Germany

**Keywords:** Infant, Child, Vocalizations, Speech-Language Development, LENA, Audio Analysis, Automatic Speech Recognition, Acoustic Analysis, Day-long Recordings, Long-form Recordings

## Abstract

Research on speech and language development has a long history, but in the past decade, it has been transformed by advances in recording technologies, analysis and classification tools, and AI-based language models. We conducted a systematic literature review to identify recently developed (semi-)automatic tools for studying speech-language development and learners’ environments in infants and children under the age of 5 years. The Language ENvironment Analysis (LENA) system has been the most widely used tool, with more and more alternative free- and/or open-source tools emerging more recently. Most studies were conducted in naturalistic settings, mostly recording longer time periods (daylong recordings). In the context of vulnerable and clinical populations, most research so far has focused on children with hearing loss or autism. Our review revealed notable gaps in the literature regarding cultural, linguistic, geographic, clinical, and social diversity. Additionally, we identified limitations in current technology—particularly on the software side—that restrict researchers from fully leveraging real-world audio data. Achieving global applicability and accessibility in daylong recordings will require a comprehensive approach that combines technological innovation, methodological rigour, and ethical responsibility. Enhancing inclusivity in participant samples, simplifying tool access, addressing data privacy, and broadening clinical applications can pave the way for a more complete and equitable understanding of early speech and language development. Automatic tools that offer greater efficiency and lower cost have the potential to make science in this research area more geographically and culturally diverse, leading to more representative theories about language development.

## 1. Introduction

Speech and language development is fundamental to human interaction, serving as the foundation for social bonding, communication and cognitive growth (e.g., Bruner, 1985; Tomasello, 2005; Vygotsky, 1978). Research on speech and language development has a long history, with studies dating back over centuries. The earliest systematic observations were conducted on individual children, often the researchers’ own offspring (e.g., Darwin, 1877; Stern and Stern, 1907; Piaget, 1952). Apart from unstructured parental reports, standardised vocabulary checklists have been widely used to measure language development since the mid-1990s (e.g., MacArthur-Bates Communicative Development Inventories/MCDI; Fenson et al., 1994; Fenson et al., 2006). Parental questionnaires, specifically MCDIs, have been adapted into many different languages (e.g., Fenson et al., 1994; Eriksson et al., 2012; https://mb-cdi.stanford.edu/adaptations.html) and have more recently been adapted for computerized-adaptive testing (e.g., Makransky et al., 2016; Mayor and Mani, 2019; Kachergis et al., 2022) and mobile app data collection (e.g., Mieszkowska et al., 2022; Muszyńska et al., 2025). Frequently, parental reports are also complemented by systematic manual annotations and analyses of audio-video recordings of children’s behaviours in experimental settings (e.g., preverbal data collection pathways as outlined in Pokorny et al., 2020) and naturalistic home recordings (e.g., for recordings, see HomeBank, VanDam et al., 2016 and Databrary, Dressler, 2015; for transcriptions of child productions see CHILDES database, MacWhinney, 2000). Manual annotation techniques, such as behavioural micro-coding and phonetic or phonological transcriptions, have been widely applied, enabling detailed analyses of expressive language functions. They require specialist training to achieve a high level of inter-rater reliability, are time- and labour-intensive, and thus, they are limited in scalability (e.g., Oller et al., 2010). When analysing speech-language functions on signal level, similar limitations apply with respect to specialised software (e.g., Computerized Speech Lab by KayPENTAX/Pentax Medical; Praat, Boersma and Weenink, 2025 or WaveSurfer, Liu, 2021). Most solutions allow for accurate visualisation of sound waveforms but do not provide a fully automated analysis process.

In the past decade, advances in recording technologies, analysis and classification tools, and AI-based language models have transformed research on speech-language development. These tools allowed researchers to advance their study of speech and language development as well as the acoustic and socio-communicative environment. To date, we have tools at hand that allow for short-term or continuous recordings across diverse contexts such as homes, nurseries, schools, and neonatal intensive care units (NICUs), offering more objective ways to record speech-language development than checklists and questionnaires that may be affected by certain bias. In recent years, we have witnessed an increase in the popularity of a variety of technical solutions to record, assess, and analyse infant speech-language development. Some of these tools are proprietary (e.g., LENA, Language ENvironment Analysis; Greenwood et al., 2011; see also Ganek and Eriks-Brophy, 2018a, 2018b for a review), others are open-source and/or free algorithms for speech diarisation (e.g., ALICE; Räsänen et al., 2021), vocal type classification (e.g., VTC; Lavechin et al., 2020) and fully automated transcription (e.g., Whisper by OpenAI; Radford et al., 2023). These tools enable scalable analyses of early vocal behaviours, expressive language development, linguistic and acoustic environments. Moreover, automated (or semi-automated) diarisation and transcription tools open new possibilities for continuous measurement of real-world behaviours that could lead to new insights into how infants interact with caregivers and siblings and how these interactions shape speech-learning processes in daily life. Overall, applying automatic tools for recording and analysing the development of child vocalisations and language opens new avenues for researchers to uncover phenomena that were previously difficult to detect using traditional methods.

To date, these tools have been used in a variety of settings and have helped to shed light on different aspects of language and communicative development, such as how the timing and frequency of caregiver responses (Weisleder and Fernald, 2013) or interaction with siblings (Laing and Bergelson, 2024) is associated with language learning trajectories (Weisleder and Fernald, 2013), and how specific environmental factors, such as household noise levels (Simon et al., 2022), background sounds (Suarez-Rivera et al., 2024), music input (Hippe et al., 2024) or digital exposure (Ferjan Ramírez et al., 2021; Brushe et al., 2024) contribute to variability in speech and language development. These tools also enable the inclusion of larger datasets collected across countries (e.g., Bergelson et al., 2023), which is important for diversifying research and asking research questions about cross-cultural differences. Moreover, they can be used to analyse how atypical developmental patterns, such as those in children with hearing loss (e.g., Altman et al., 2020; Aragon and Yoshinaga-Itano, 2012; Josvassen et al., 2024; Lee and Ha, 2024), genetic conditions (e.g., Marschik et al., 2017; 2022; Pokorny et al., 2016; 2022), autism (e.g., Oller et al., 2010; Warlaumont et al., 2014, see review in Putnam et al., 2024), and developmental delays (e.g., Oller et al., 2010), impact early language learning, providing critical insights for intervention strategies. However, the introduction of these tools also brings with it new challenges. Many current tools or algorithms are insufficiently flexible to account for diverse situational, linguistic and cultural settings, particularly in low-resource environments (Cristia et al., 2024). Moreover, speech recognition tools still struggle with infant vocalisations and child speech, which include a mix of prelinguistic vocalisations, proto-words and target-language utterances. To enhance the utility of modern technology in the field, it is essential to examine the scope of its existing application—including the populations, cultural contexts, and environments studied, as well as its limitations. This will help identify opportunities for improvement.

Here, we conduct a systematic literature review to identify recently developed (semi-)automatic tools for studying speech-language development and learners’ environments in infants and children under the age of 5 years. We overview how these methods have been applied, including the country of publication, the spoken language studied, and the settings in which the methods were used. We review if participants were recruited from physiological/typical or clinical cohorts. Furthermore, we report what recording equipment and tools were used for data analysis.

In the narrative synthesis of results, we systematically evaluate the strengths, limitations, and potential of existing tools and discuss their impact on speech-language research in infancy and early childhood, while providing examples of studies. We begin with tools for acoustic analysis that commonly require labour-intensive manual pre-processing. We go on to examine the LENA system, the most used tool for both recording and analysing speech-language development. This is followed by summarising alternative approaches for audio recording and analysis, considering their impact, potential and limitations for future applications. In the Discussion section, we embed findings from the systematic review in the broader context of theory and practice in developmental science, presenting newly emerging technological solutions and possible research directions that can advance our knowledge of speech-language development. Highlighting opportunities for innovation and expansion, we advocate for developing more inclusive, ecologically valid and reliable approaches for investigating language acquisition across diverse contexts, including various clinical conditions and cultural groups.

## 2. Methods

To systematically review the state-of-the-art methodologies for capturing and analysing speech and language, we conducted database searches following PRISMA guidelines (Page et al., 2021). The search strategy used the specific terms outlined below and was limited to peer-reviewed publications in English, excluding grey literature. The final search was completed on December 9, 2024, and included studies published from the year 2000 onward. The datasets generated by the survey research and analysed during the current study are available in OSF: https://osf.io/xge3s/?view_only=e94b350bbe754a23841a10b1d7f8f887. The code for generating figures is available on GitHub: https://github.com/kpatsis97/Cost_review.

### 2.1. Search strategy

The search strategy was tailored to PubMed and EBSCOhost, using database-specific search terms:

- PubMed: (((infan*[tiab]) OR (child*[tiab])) AND ((vocal*[tiab]) OR (speech[tiab]) OR (daylong audio recordings[tiab])) AND ("computational analysis"[tiab] OR "automatic classification"[tiab] OR "automatic measurement"[tiab] OR "acoustic analysis"[tiab] OR "audio analysis"[tiab] OR "automatic speech recognition"[tiab] OR “LENA”[tiab]));
- EBSCOhost: AB (((infan*) OR (child*)) AND ((vocal*) OR (speech) OR (daylong audio recordings)) AND ("computational analysis" OR "automatic classification" OR "automatic measurement" OR "acoustic analysis" OR "audio analysis" OR "automatic speech recognition" OR “LENA”)).

### 2.2. Selection criteria

#### 2.2.1. Title-abstract review

The exclusion criteria for this first selection phase were: (a) publication before the year 2000; (b) systematic reviews, meta-analyses or dissertations; (c) majority of participants older than 5 years; (d) no reference to the recording and/or analysis using semi-automatic or automatic methods. The first 100 abstracts (22.5 %) were independently double-screened by two authors (ZL, MD). The initial kappa value for interrater reliability was к = .69. All disagreements were resolved by consensus. In the second step, 50 additional abstracts were independently screened by the same authors, improving the kappa to к = .74. Following this substantial agreement, the remaining articles were split for review between the two authors.

#### 2.2.2. Full-text review

**Fig. 1.**
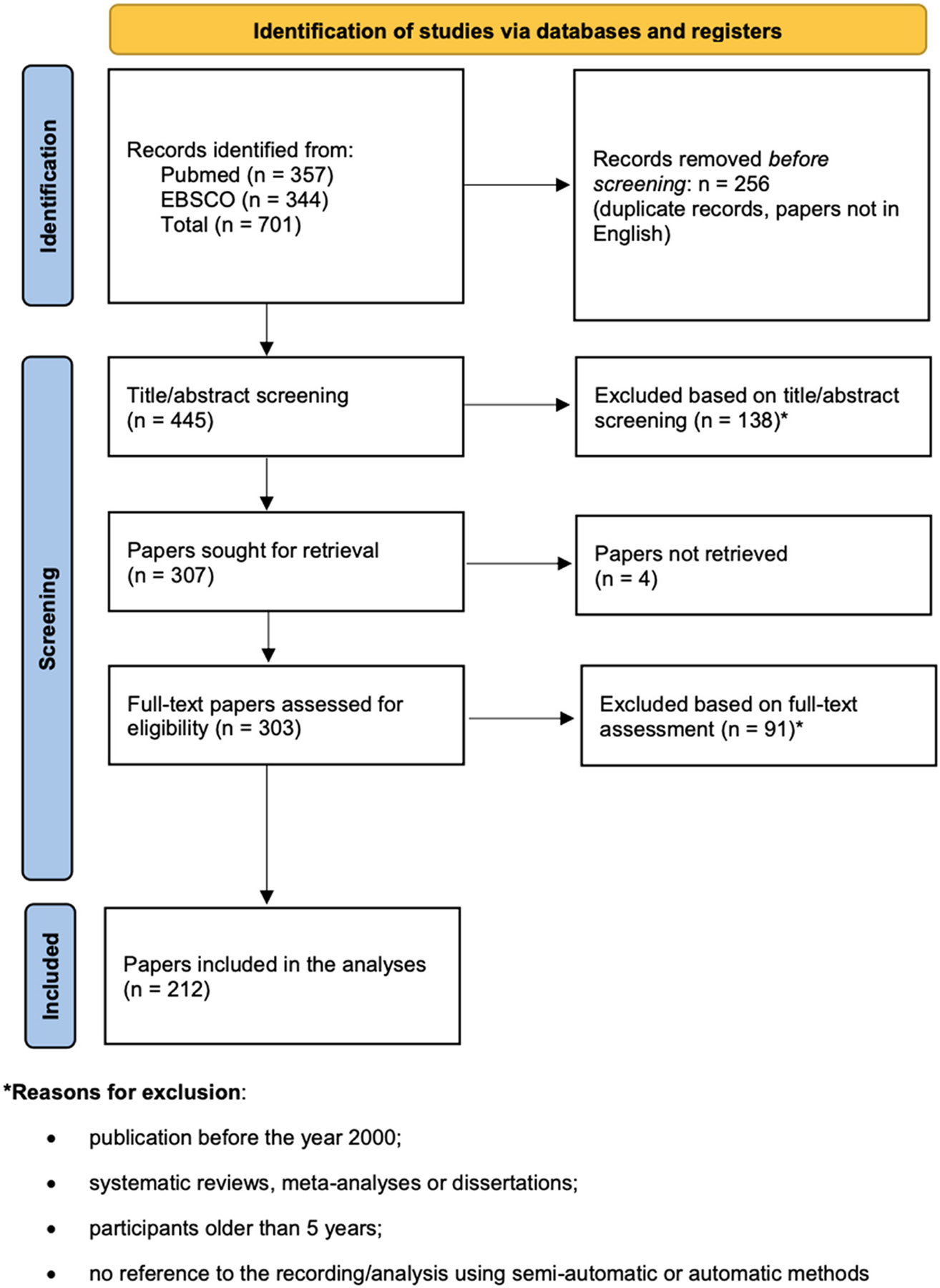
PRISMA Flow diagram.

Full texts of all studies that passed the title–abstract review phase were retrieved and split between three authors (ZL, AC, MD), who applied the same exclusion criteria as in the previous article selection phase (A) (see Fig. 1).

#### 2.2.3. Study selection

The systematic search strategy yielded a total of 701 publications. After removing duplicates and papers written in a language other than English, 445 publications proceeded to the first screening stage (A). The title–abstract review eliminated 138 publications, leaving 307 for the full-text review (B). Out of those 307, four papers could not be retrieved, leaving 303 publications retained for data extraction and in-depth review. During the full-text review, a further 91 publications were excluded, again applying the above-mentioned criteria, resulting in 212 publications included in the review (see Fig. 1).

## 3. Results

**Fig. 2.**
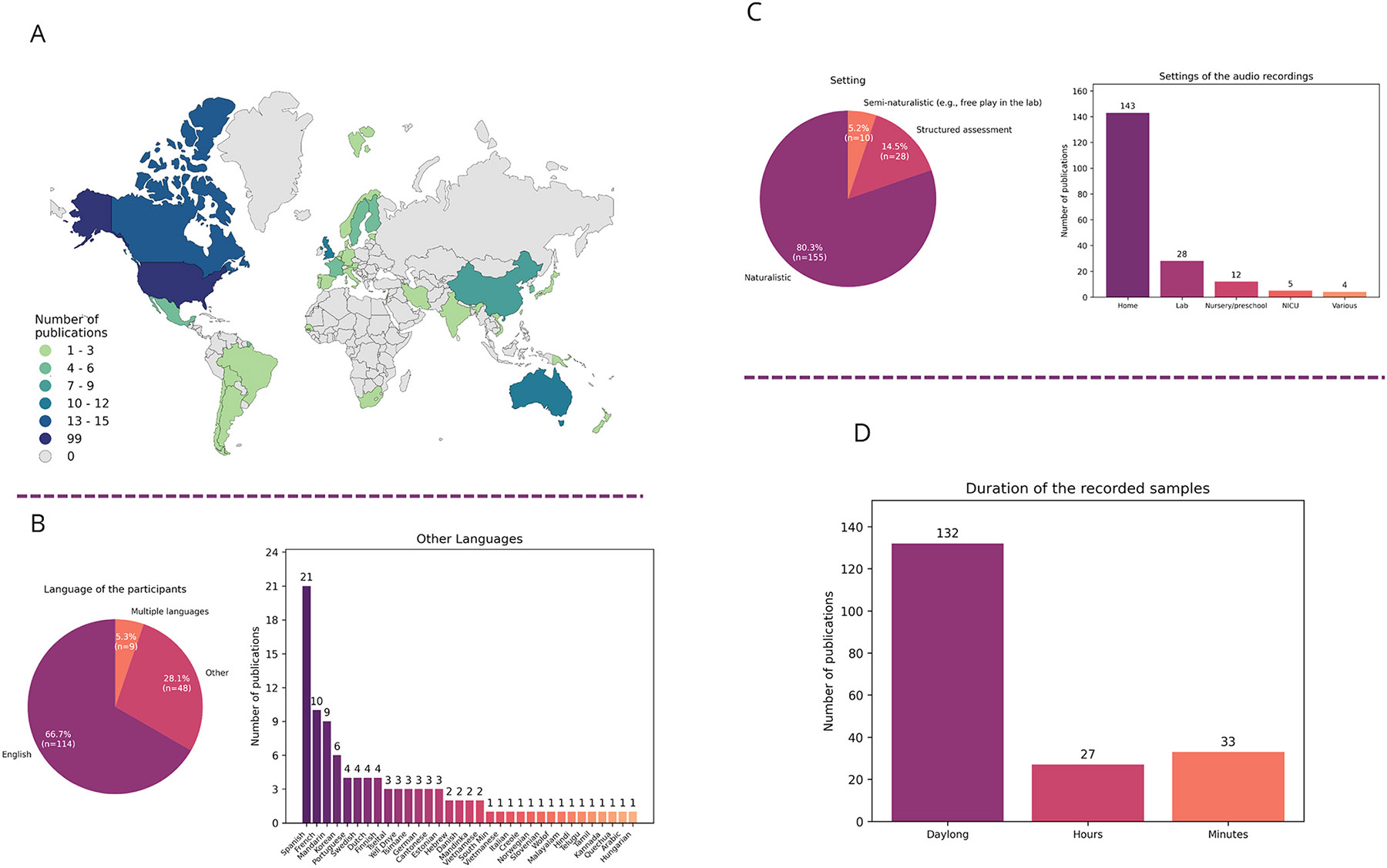

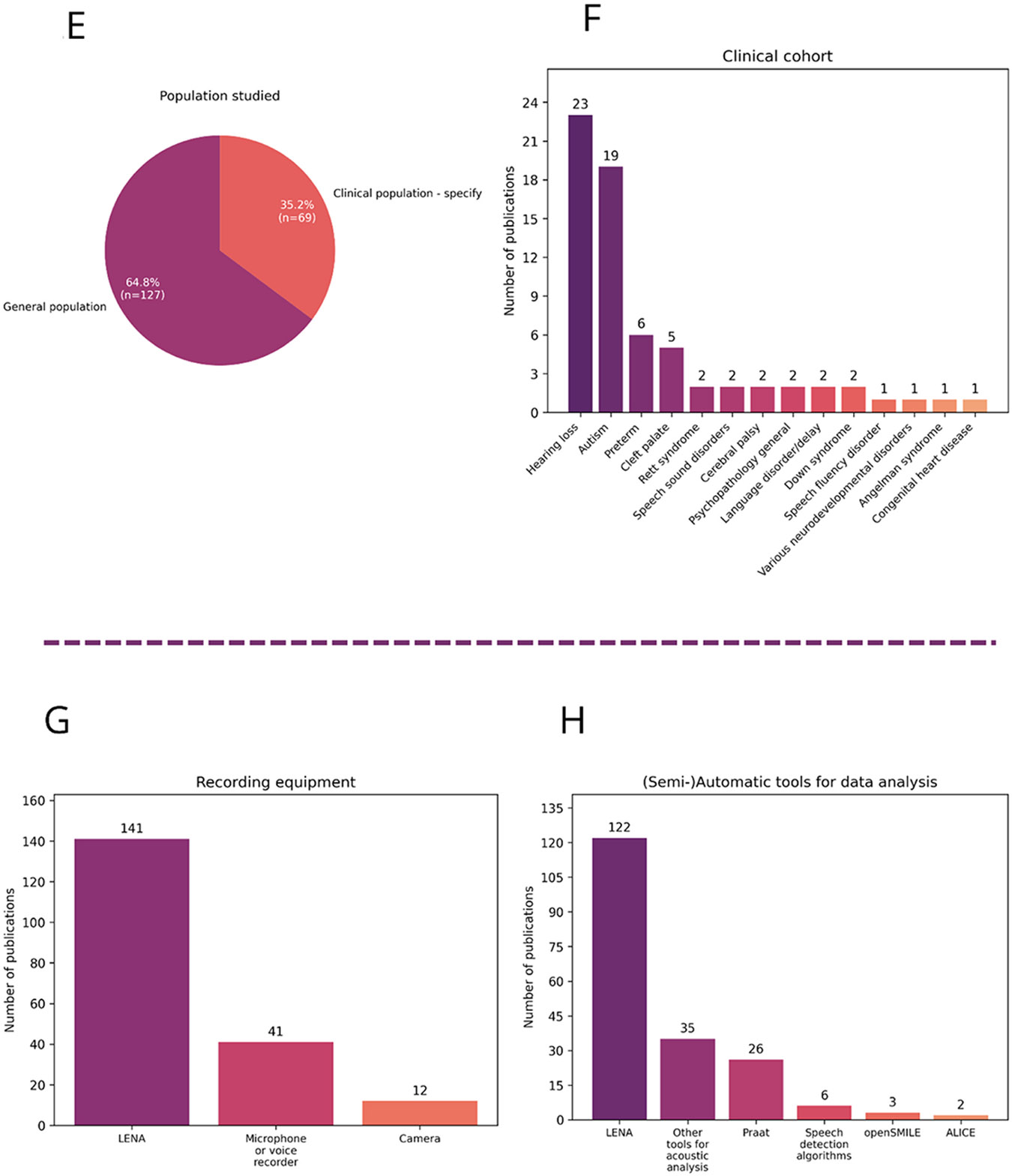
Overview of the results based on the number of publications in a given category.

**Fig. 3.**
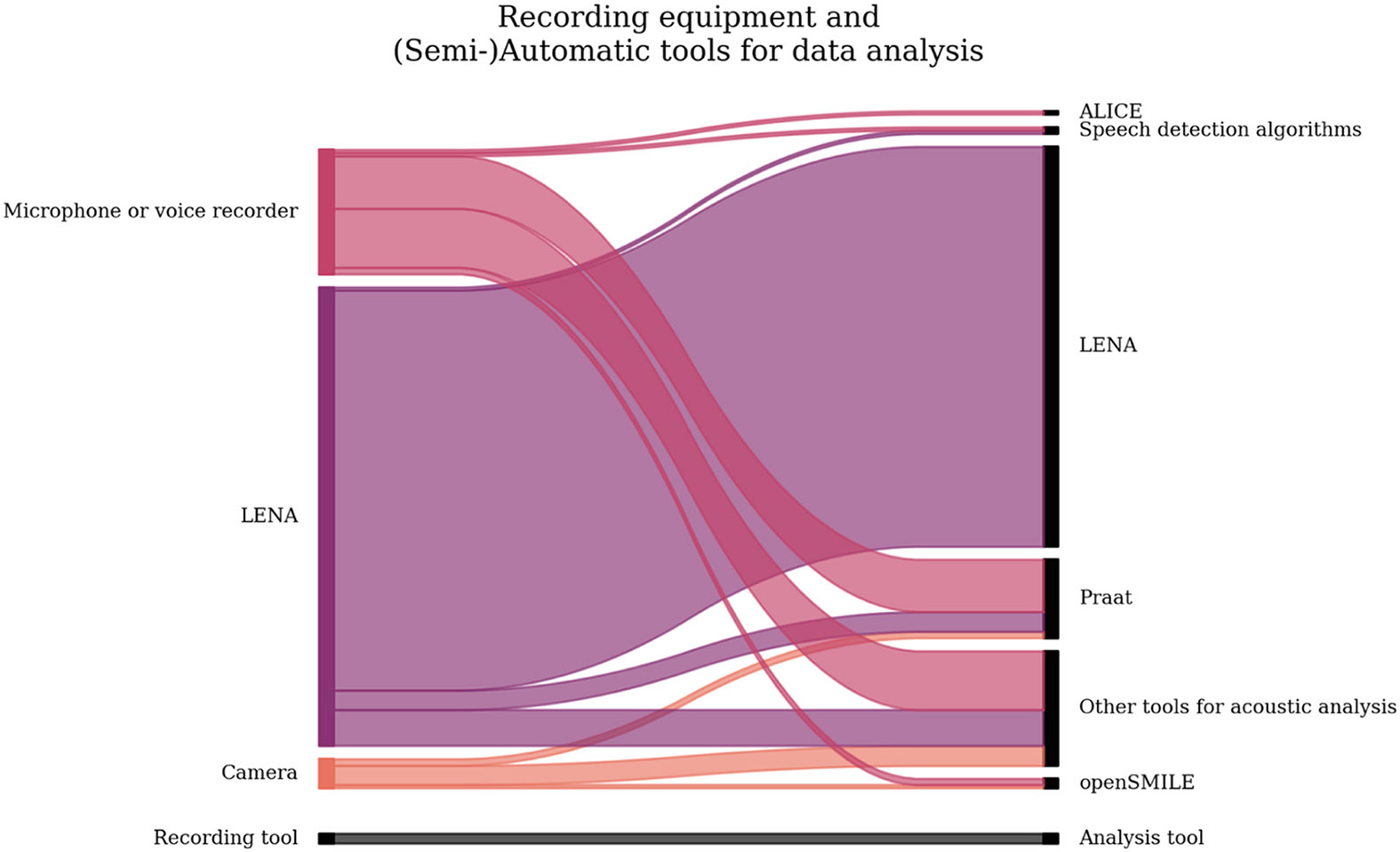
The Sankey diagram shows the tools used for recording (left) and the corresponding tools used for data analysis (right). The width of the band indicates the number of publications with each combination.

**Fig. 4.**
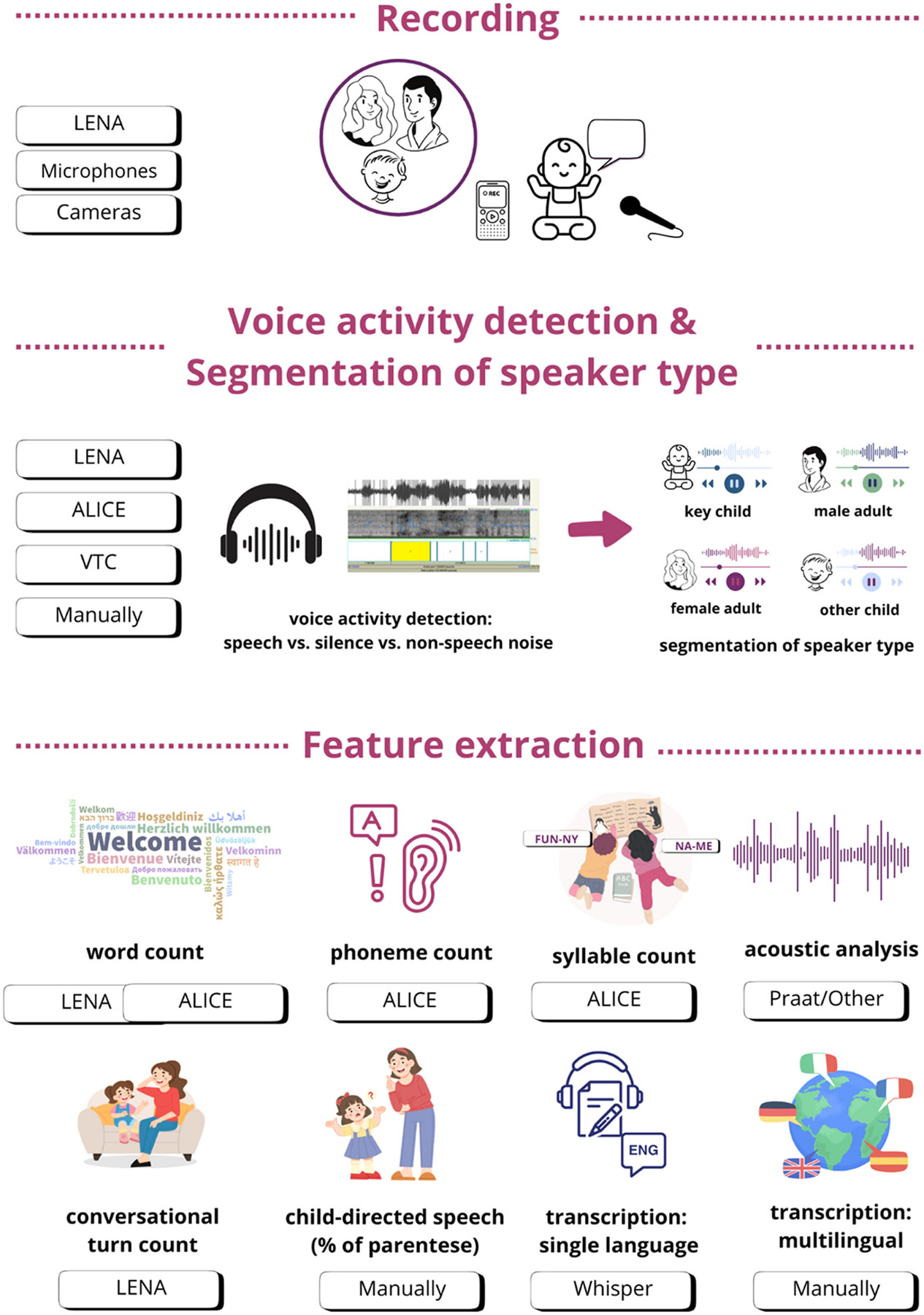
Overview of the recording and analysis process and tools. LENA: Language Environment Analysis; ALICE: Automatic LInguistic unit Count Estimator; VTC: Voice Type Classifier; Whisper: a neural net by OpenAI; Praat: open source software.

Our systematic literature review was focused on recently developed (semi-)automatic tools for studying speech-language development and learners’ environments in infants and children under the age of 5 years. Regarding participants’ geographic location and language, English-learning children in the U.S. were the most intensively studied (Fig. 2A, B). The majority of studies (80.3 %) were conducted in naturalistic settings such as participants’ homes, nurseries and preschool classrooms (Fig. 2C), mostly recording longer time periods, even across the entire day (Fig. 2D). A fifth of the screened studies (19.7 %) were conducted in semi-naturalistic settings, recording free play in the lab or some form of structured assessment over shorter periods of time (hours or minutes in contrast to daylong recordings, Fig. 2C, D). In the context of more vulnerable and clinical populations, most research so far has focused on children with hearing loss and children with (elevated likelihood of) autism (Fig. 2E, F). The search revealed a few major tools for studying early vocal/speech production and linguistic environments. LENA has been the most used solution for recording, with microphones/voice recorders and cameras being less frequent tools (Fig. 2G). LENA has also been the most widely used tool for analysing (pre)linguistic development (Fig. 2H). Other approaches for analysis were primarily non-commercial and custom-made by research groups. Fig. 3 presents the combination of tools used for recording and analysing data and Fig. 4 shows an overview of the recording and analysis process and tools.

### 3.1. Narrative synthesis of results

Here, we summarise key findings and examples of studies that applied (semi-)automatic tools in studying speech-language development in infancy and early childhood, starting with tools for acoustic analysis that were some of the first semi-automatic approaches to analyse speech-language development, still requiring significant efforts related to manual preprocessing of recordings. Then, we describe findings related to LENA-based studies and the limitations of this approach. Finally, we present recording and analysis alternatives to LENA.

### 3.2. Acoustic analysis

Some of the first initiatives to automate the analysis of speech-language development were focused on developing specialised software for acoustic analysis. Acoustic analysis of audio signals and extraction of key features, such as speech sounds and words, is necessary to conduct any higher-level analyses of speech production. Such programs provide visual displays of audio signals such as waveforms, amplitude spectra and spectrograms. They also provide quantitative analysis derived from these acoustic waveforms and spectrograms, which can be valuable for assessing speech and language development and are widely used in research and clinical practice in the fields of speech pathology and audiology for diagnosis and monitoring treatment progress (Segura-Hernández et al., 2019; Coelho et al., 2015; 2016; Knight et al., 2016; Glaspey and Macleod, 2010; Chang et al., 2002). Acoustic analysis was used, for example, to investigate the impact of age at cochlear implantation on vocal development in children (e.g., Knight et al., 2016), the effects of vocal rehabilitation on voice acoustics in children with cleft lip and palate (Segura-Hernándeza et al., 2019) and the changes in voice physiology after surgical correction in infants with congenital heart disease (Joo et al., 2015). Some studies used proprietary software-hardware solutions such as the Computerized Speech Lab (KayPENTAX/Pentax Medical, e.g., Smith et al., 2008; Goldfield, 2000) that can include software modules such us Multidimensional Voice Program (MDVP, for detailed analysis of voice quality by measuring parameters like jitter, shimmer and harmonic-to-noise ratio, Coelho et al., 2016; Knight et al., 2016; Joo et al., 2015), Multi-Speech (for acoustic analysis and speech signal processing, Niwano and Sugai, 2002) and Real Time Pitch (focuses on pitch tracking and provides visual feedback for pitch variation, Coelho et al., 2016).

Other studies relied on free and open-source software such as Praat (Boersma and Weenink, 2025), which supports pitch tracking, spectrogram analysis, and formant measurement. It can also be used for manual annotation of vocalisations (León et al., 2023; Mahmoudian et al., 2019; Mealings and Demuth, 2014). Other examples include TF32 (Milenkovic, 2018) for time-frequency analysis, which displays the acoustic waveform along with pitch and sound spectrogram (e.g., Yoo et al., 2019) and WaveSurfer (Liu, 2021). This methodological approach allows for in-depth phenotyping of acoustic properties and voice quality in typically and atypically developing children. However, these tools have been minimally tested using large datasets or daylong recordings. While some studies have applied tools like PRAAT for large-scale acoustic analyses (Ritwika et al., 2020), systematic validation of pitch estimates in long-form recordings remains limited. Further, because many acoustic features, such as F0, are meaningful only within voiced speech, researchers must carefully consider the theoretical foundations of speech signals to avoid misinterpreting acoustic analyses, especially in long-form recordings where a diverse range of vocalisation contexts arise. Additionally, while automated tools could potentially facilitate acoustic analysis (see Pokorny et al., 2022, for an example of combining manual annotation of vocalisations with automatic classification based on acoustic features), they do not replace the need for human oversight in selecting and segmenting relevant portions of audio, a crucial step before meaningful interpretation can occur. This limitation constrains the potential for large-scale applications.

### 3.3. LENA – currently the most common tool for recording and analysing speech-language development

An important milestone in automating the measurement of child speech and language environment was the development of the proprietary system Language Environment Analysis (LENA) (Greenwood et al., 2011). To date, LENA is the most commonly used tool (Fig. 2G, H), so we begin with a more detailed overview of its features, which also illustrates the requirements of researchers in the field and the challenges faced by new tool developers. LENA is a commercial tool for audio recording, language measurement, and basic analysis designed for long-form recordings. The system consists of a small audio recorder with a single microphone, which is made to be placed in a chest pocket sewn into children’s clothing (vest, t-shirt, overalls) made by LENA for this purpose. Once the audio has been collected, the software analyses the recordings and provides a numerical and graphical data output. First, the LENA algorithm classifies speech into speaker type (female adult, male adult, key child, and other child) and non-speaker classes (noise, television, and silence). For speech recorded from the key child (the child wearing the audio recorder), LENA uses algorithms to identify and exclude crying or vegetative sounds. This ensures that only linguistically relevant vocalisations from the child are included in the output. The final output provides researchers with numerical data on various features captured from the linguistic input recorded by the LENA device, including adult word count, conversational turn count, and child vocalisation count. Conversational turns are defined as pairs of adult-child or child-adult vocalisations separated by no more than five seconds. Research using LENA has successfully contributed to a substantial body of work on infant and child language development, both for exploratory research purposes (e.g., Laing and Bergelson, 2024; Orena et al., 2020; Weisleder and Fernald, 2013) and intervention programs for children (e.g., Cunha et al., 2024; Joseph et al., 2022; Rowe et al., 2023; Suskind et al., 2016). Exploratory research has used LENA to investigate a variety of linguistic features of children’s environments, for example, the language input to preterm infants (Caskey et al., 2011) and to children with diverse abilities and neurodivergent profiles (e.g., Aragon and Yoshinaga-Itano, 2012; Dykstra et al., 2013; Irvin et al., 2013; Oller et al., 2010; Thiemann-Bourque et al., 2014). Research with LENA has also been used to measure the amount of language exposure children receive in monolingual English-speaking homes (e.g., Bergelson et al., 2019) and bilingual homes (e.g., Marchman et al., 2017; Orena et al., 2020) in North America. Researchers have also implemented parent-focused interventions using LENA, such as parental interventions in low SES samples (e.g., Cunha et al., 2024; Joseph et al., 2022; Rowe et al., 2023; Suskind et al., 2013). In addition to studies examining the speech children hear from their environment, researchers have used LENA to explore the interaction between the language input children receive and the language output they produce. For example, studies have investigated the relationship between the number of conversational turns between adults and children and the child’s overall word count (Weisleder and Fernald, 2013), as well as adult response rates to both speech-like and non-speech vocalisations (Warlaumont et al., 2014). Researchers have also used LENA to focus specifically on infants’ vocalisations, such as comparing the amount of speech-like vocalisations to crying vocalisations (Oller et al., 2021).

### 3.4. LENA research in languages other than English

LENA has been predominantly used in monolingual English-speaking communities in North America. The original dataset used to train LENA’s algorithms included over 65,000 hours of recordings from over 300 monolingual English-speaking families raising infants between 1 and 42 months in North America (Gilkerson and Richards, 2008a). Since then, LENA has been used with a wide range of languages, and numerous papers report on the accuracy of their algorithms: Hebrew and Arabic (Levin-Asher et al., 2023), Mandarin Chinese (Zhang. X. et al., 2024), Danish (Josvassen et al., 2024), Slovenian (Ferjan Ramírez et al., 2024), Shanghainese-Mandarin (Gilkerson et al., 2015), Vietnamese (Ganek and Eriks-Brophy, 2018a, 2018b), French (Canault et al., 2016), Dutch (Bruyneel et al., 2021), Swedish (Schwarz et al., 2017), Spanish (Weisleder and Fernald, 2013) and Italian (Bastianello et al., 2024). A recent systematic review of studies focusing on the validation of LENA across various languages found that, while some LENA outputs had moderate to high accuracy across languages (e.g. child vocalisation counts), others did not (conversational turn counts) (Cristia et al., 2020). Moreover, previous validation studies have typically focused on a specific corpus where participants share similar age ranges and languages. This makes it challenging to determine whether any differences in results are due to variations in how the corpus was annotated by researchers, or if LENA’s accuracy is influenced by the specific characteristics of the population, such as age range and language. For instance, accuracy assessments of LENA vary widely across studies. Canault et al. (2016) considered LENA to be sufficiently reliable for adult speakers according to their reliability score (*r* = .64), whereas Meera et al., 2025 showed similar reliability for adult speakers (*r* = .62) but concluded there was still scope for further improvement (Meera et al., 2025). These discrepancies highlight the subjectivity in determining whether a given level of accuracy is ‘good enough’. To validate LENA reliably for use in multiple languages, it is essential for researchers to develop a standardised validation process applicable to diverse datasets. Beyond accuracy metrics, validation can also be approached at the level of findings rather than individual classifications. Some errors may not systematically bias results, meaning that even an imperfect classifier may still be useful for capturing broader patterns (e.g., Ritwika et al., 2020). A robust validation framework should, therefore, consider both quantitative accuracy and the broader implications of classification errors on research conclusions. Such an approach to validation could also be applicable to other tools designed to analyse daylong recordings.

### 3.5. Limitations of LENA

While studies using LENA have contributed to a substantial body of research that has advanced early language development research, LENA can be costly for research labs. To start a project using two recorders over 6 months, the costs could be up to $20,000, including equipment and subscription costs to LENA ((LENA Research Foundation, n.d) prices as of February 2025). Therefore, research institutions with fewer financial resources may lack the funds to conduct such research, limiting its applicability. Furthermore, new versions of the LENA analysis component require researchers to upload participants’ recordings to servers based in the U.S. In some countries, particularly in the European Union, legal restrictions, such as those under GDPR, pose challenges for data transfers to non-European sites, sometimes leading to the rejection of research proposals based on legal rather than ethical considerations. Navigating these legal requirements involves substantial administrative effort, which can limit the feasibility of such research.

The level of performance for some of LENA’s outputs relating to infant vocalisations has been critically discussed, e.g. highlighting poor performance for the key child’s vocalisation recall, with one study finding LENA captured only 50 % of the key child’s vocalisations identified by human annotators (Cristia et al., 2020). In addition, while LENA can distinguish between speech-like vocalisations and non-speech vocalisations (e.g., crying), its algorithms cannot provide more detailed classifications, such as distinguishing between canonical and non-canonical babbling or between different levels of crying (e.g., crying for different emotional needs). Thus, researchers using LENA to collect and identify instances of infant vocalisation often need to analyse those vocalisations in further detail. This can be achieved either manually (e.g., Soderstrom et al., 2021) or by extracting detailed acoustic features using OpenSMILE (Eyben et al., 2010). While these features provide valuable insight, they are typically only a starting point for a deeper analysis. Advanced algorithms, such as models designed to detect infants’ emotional responses (e.g., ZhuParris et al., 2021), are often required to interpret the data for the studied research questions adequately. Between the limits to the accuracy and detail of the LENA system’s automatically generated labels, the high cost of the system, and the proprietary algorithms, researchers are increasingly turning to alternative audio recording devices and analysis software.

### 3.6. Recording and analysis alternatives to LENA

In recent years, alternative audio recording and analysis methods have been employed (e.g., Casillas et al., 2020; Cristia et al., 2023, see also Lavechin et al., 2025). Devices for recording purposes include USB "spy" recording devices (e.g., Cristia et al., 2023; Scaff et al., 2024; Caunt and Abu-Zhaya, 2024) and Olympus recorders (e.g., Casillas et al., 2020; Scaff et al., 2024) and the average prices are in the low-to-moderate range (starting from $20–70 for a basic voice recorder). While most of these devices do not come with their own software for speech analysis, researchers engaged in this line of work have been investing in building open-source alternatives to the closed-source LENA speech processing algorithms. Such open-source alternatives can provide researchers with similar outputs to those provided by LENA.

Firstly, the voice type classifier (VTC; Lavechin et al., 2020) was created with the purpose of classifying audio segments into speaker categories similar to LENA, for example, *female adult*, *male adult*, *key child* (the child wearing the recording device), and *other child*. Building upon this, the Automatic LInguistic unit Count Estimator (ALICE; Räsänen, 2021) was created using the VTC, allowing researchers to measure additional metrics in their data, such as the number of words, syllables and phonemes produced by adult speakers. These additional features and the ability to fine-tune them are essential for enhancing the algorithm’s usability across different languages. For example, estimating word count can be challenging in languages where acoustic patterns and language-specific lexical entries do not follow the same sentence structure or acoustic patterns. By counting phonemes and syllables in parallel with words, ALICE allows researchers to use the algorithm across different languages. Both the VTC and ALICE have been shown to either outperform LENA or achieve similar performance, although the different systems make different trade-offs in terms of precision versus recall (Lavechin et al., 2020; Räsänen, 2021). However, researchers need programming proficiency in Python to use ALICE and the VTC effectively. Without sufficient experience in Python or technical support, researchers may encounter challenges, which could create barriers and limit labs’ ability to automate the processing of audio data.

Further challenges arise in analysing infant vocalisations. Currently, no widely available algorithms provide detailed automated speech analysis of day-long audio files (e.g., 16-hour recordings) beyond simple key-child segmentation of vocalisations. While algorithms like ALICE and VTC show promise for day-long recordings through speaker diarization, they were not trained to categorise features of infant vocalisations. Although researchers have employed feature extraction tools such as OpenSMILE (Eyben et al., 2010) on shorter audio segments (up to 10 seconds) to generate statistics on infants’ emotional states, such as cry detection (ZhuParris et al., 2021; Micheletti et al., 2023) and the classification of babbles (e.g., canonical vs non-canonical, Fell et al., 2003; Yeh et al., 2019; vocant vs squeal vs vowel, Warlaumont et al., 2010), there are no open-source tools that provide comprehensive labelling of infant vocalisations across different stages of speech-language development (cooing, babbling, proto-words etc.). Further, advancing current algorithms to classify infant vocalisations – e.g. distinguishing canonical from non-canonical babbles – within a single program rather than requiring multiple different tools (e.g., one software for audio preprocessing and another for classification) would be a significant improvement. These challenges highlight the need for continued development in algorithms for recognising infant vocalisations, driven by several factors, including the highly variable and context-dependent nature of infant vocalisations, as well as the need to create flexible tools that can analyse a broad range of vocalisations across age groups and developmental stages.

There is significant potential for advancements in this area, which could be achieved through increased collaborations between speech technologists and researchers collecting data with infants. This collaboration would be crucial in developing more effective algorithms and tools. A key factor in driving these advancements would be the establishment of a culture of data sharing, which will enable better training of classifiers and the creation of open-source tools that are tailored for the analysis of infant vocalisation (VanDam et al., 2016). To facilitate this, developing multi-site recording efforts and ensuring open data sharing across research groups will be crucial for building a robust foundation for the analysis of infant speech.

Other important limitations of the currently–available automatic tools that need to be addressed include the issues with the correct classification of various child speakers present in the recording. This is crucial for research conducted in more noisy environments with multiple speakers present at the same time, such as in a nursery or preschool. Similar concerns are related to capturing the simultaneous speech of multiple speakers that consists of many overlapping vocalisations. Additionally, these tools must be adapted to ensure accessibility for low-income, linguistically and culturally diverse, and neurodiverse populations, making the methodology more inclusive and widely applicable.

## 4. Discussion

In this review, we aimed to identify key tech-enhanced tools for studying speech-language development in infants and children under 5 years of age. We present an overview of methods that allow for investigating early speech-language skills as well as learners’ environments. We review studies focusing on different language acquisitional aspects such as acoustic analysis of audio signals, frequency of vocalisations, or conversational turns. In our discussion, we highlight the existing sampling bias regarding geographical regions and linguistic groups. We point out the underrepresentation of neurodiverse and clinical groups in the research landscape and discuss how technological and utility limitations could hamper broader and more inclusive research practices.

### 4.1. Sampling bias and consequences for understanding language acquisition

The majority of research on speech-language development has been conducted in the Global North, with participants from the U.S. and Western Europe. This geographical bias has systematically influenced theories on language development. Some theories have already been challenged by data from daylong recordings collected in non-WEIRD populations (Henrich et al., 2010). For example, research on children living in the U.S. has suggested that child-directed speech produced by adults is a key type of vocal input that significantly impacts children’s language development and children who are exposed to more child-directed speech are developing faster-growing linguistic capacities (e.g., Weisleder and Fernald, 2013). However, recent findings point out that in some cultures, children are infrequently spoken to (Casillas et al., 2020), the quantity of child-directed speech is relatively low (Bunce et al., 2024), and the association between children’s vocal output and input of vocalisations produced by other children is stronger than the association with vocalisations produced by adults (Cristia et al., 2023). Thus, the structure of home life in various cultural contexts relates to exposure to child- and adult-directed speech on a daily basis (e.g., Bunce et al., 2024; Casillas et al., 2020). Another prominent theory based on data from children in the U.S. suggests that children of families from lower socioeconomic status (SES) backgrounds are exposed to less language input, which impacts their speech-language development (e.g., Hart and Risley, 2003). Yet, a recent study conducted across six continents, which also used daylong recordings to examine language input and children’s vocal output, found no significant association between SES and children’s input quantity or vocal production (Bergelson et al., 2023), showcasing how the use of novel tools can challenge monocultural theories on speech and language learning.

Automatic tools for processing (daylong) recordings and speech recognition in real-world settings can also help broaden existing theories to better account for factors that contribute to speech acquisition, such as, for instance, capturing the influence of a wider diversity of caregivers (Katus et al., 2024), siblings (e.g., Laing and Bergelson, 2024) and other peers (e.g., Perry et al., 2018) on the development of early conversational turns. Automatic tools that offer greater efficiency and decreased cost compared to manual annotation and transcription of recordings have the potential to make science in this research area more diverse. Moreover, they facilitate tracking infants’ daily electronic media exposure as a component of their language environment and its impact on their vocalisations (e.g., Ferjan Ramírez et al., 2022; Brushe et al., 2024). Taken together, these recent findings on language acquisition highlight the value of research that spans not only different cultures, but also different contexts and household structures.

### 4.2. Future challenges and opportunities

Making research on speech-language development more accessible for global applications and clinical populations is a multifaceted challenge requiring technological innovation, methodological adaptation, and ethical vigilance. Below, we discuss potential pathways and considerations organised by key challenges and opportunities.

A significant limitation in current research is the overrepresentation of studies focused on typically developing English-learning children in the U.S. Findings from these studies, while informative, do not capture the diversity of early language development on a global scale (see Singh et al., 2025 for a broader discussion). Expanding research to include populations from diverse linguistic, cultural, geographical and socioeconomic contexts is critical. However, practical barriers such as funding, training, and infrastructure often impede these efforts, particularly in low- and middle-income countries (LMICs). Establishing respectful and collaborative partnerships with local institutions and investing in capacity-building initiatives can help overcome these challenges (e.g., Léon et al., 2024). Similarly, the efforts to develop free and open-source tools can also result in broader use across different research sites. Furthermore, the dominance of English-speaking participants in language development research (Fig. 2A) creates a feedback loop that perpetuates the scarcity of data from other languages. Insufficient data hinders the development of tools for under-represented languages. Further, the limited applicability of these tools in non-English languages can discourage researchers from collecting new data. To break this cycle, strategic efforts to collect and share recordings in diverse languages are essential (see the excellent example in Bergelson et al., 2023). Collaborative cross-linguistic research efforts and shared repositories of annotated data are essential steps towards building a more comprehensive dataset that reflects the diversity of global languages. Another step is training initiatives accessible to diverse groups of researchers – with a particular emphasis on involving research groups from LMICs – focused on cross-linguistic validation of various algorithms dedicated to infant/toddler data and specialised programming workshops. Finally, new funding opportunities supporting multi-site collaborations would allow for minimising the practical barriers.

In clinical contexts, most research has focused on populations such as children with hearing loss and those with (elevated likelihood of) autism (Fig. 2E, F, see also Putnam et al., 2024 for a review of LENA-based studies focused on autism). Broadening the scope to include other clinical and/or vulnerable populations, such as children with developmental language disorder and Down syndrome, is necessary to understand the full spectrum of challenges related to early language development. Automatised AI-supported speech analysis might augment the clinical diagnosis and thus support earlier and more reliable identification and differentiation of different types of speech disorders (e.g., phonological, dysarthria, childhood apraxia of speech). However, obtaining patient data for algorithm-training purposes poses unique challenges due to the sensitive nature of medical data. For this reason, more consortia-based collaborations with predefined rules of data sharing across sites are necessary to develop automatic tools to accommodate the needs of specific clinical populations.

Technological limitations also restrict the accessibility of daylong recording studies. For example, the Language Environment Analysis (LENA) system, widely used in the field, faces several challenges in adapting to new languages and contexts. LENA’s algorithms were trained on English recordings, and validation efforts in other languages have shown mixed results, underscoring its limited generalisability. Open-source alternatives offer a promising avenue but often require programming expertise, creating barriers for researchers and clinicians without technical training. Additionally, cloud-based systems cannot be easily used in many European countries due to data privacy regulations – this issue is especially pressing for clinical research, where safeguarding sensitive data is paramount. Decentralised or localised data-processing solutions could mitigate these challenges and ensure compliance with regional regulations. Simplifying the setup and usability of open-source tools through community-driven platforms and shared resources could further enhance their adoption.

While partial solutions exist for sharing long-form audio data as well as annotations and tools (e.g., HomeBank, VanDam et al., 2016), data sharing remains a major challenge in the field, particularly when balancing open science principles with ethical and legal obligations to protect participants’ privacy. Sharing annotated recordings on such platforms, along with organising thematic engineering challenges such as ComParE (Computational Paralinguistic Challenge; Schuller et al., 2020), can accelerate algorithm development, but this must be done in a way that respects participants’ rights and adheres to ethical guidelines, as emphasised by Cychosz et al. (2020). Innovative approaches such as federated learning - when the algorithm travels, not the data (see example for pediatric care in Rb-Silva et al., 2023), allow data analysis without the transfer of raw data and could be a promising direction for certain research scenarios. In addition, anonymisation techniques (such as sharing annotations, transcripts and output files rather than raw audio data) and secure data-sharing protocols are essential to ensure ethical and responsible research practices.

Data coming from daylong recordings can facilitate the development of computational models of early language acquisition (Dupoux, 2018; Lavechin et al., 2022). Daylong recordings provide realistic input data in ecologically valid conditions that can be used to train AI algorithms. The approach of reverse engineering language development, i.e. building computational systems that can be trained on realistic input data to mimic the process of infant language acquisition, can provide us with scientific insights into human language learning (Dale et al., 2022). In addition, it allows for the creation of better artificial language learners (Dupoux, 2018; Lavechin et al., 2022).

Whilst naturalistic recordings offer valuable insights into real-world language exposure, it is important to consider whether they are sufficient on their own or if experimental approaches remain necessary. Naturalistic recordings can capture authentic interactions between children and others and provide data to input into computational models, yet the data can be limited in recognising causal relationships or systematically manipulating variables. Thus, researchers could integrate experimental methods to test specific hypotheses, using a mixed-methods approach where naturalistic data informs experimental design and vice versa. This combination could lead to a more comprehensive understanding of early language acquisition.

A recent breakthrough in minimising time- and labour-intensive tasks in speech development research comes from developments in open-source automatic speech recognition (ASR) models, such as the Whisper algorithm developed by OpenAI (Radford et al., 2023). It is designed to effectively handle transcription in many languages (although not when multiple languages are spoken in a single recording) and translation tasks, using a transformer-based architecture trained on diverse datasets of speech recordings and their transcriptions. Whisper emphasises robustness over a wide range of accents, background noise and languages, making it suitable for real-world applications. However, children’s speech is characterised by high variability in pitch, articulation, and speech patterns due to developmental differences (e.g., Li, 2012; Redford, 2014), so it can present unique challenges to automatic speech recognition algorithms, which may be built with limited infant/child-centric training data. Nevertheless, preliminary reports suggest that Whisper is a promising tool for recognising children’s speech recorded in challenging environments – for example, in noisy school environments (Southwell et al., 2024) or spoken by children who are not native English speakers (Jain et al., 2024).

Finally, improvements in data collection and analysis techniques are critical to capturing the complexity of language environments. Emerging research highlights the importance of contextual factors, such as exposure to electronic media (Ferjan Ramírez et al., 2022; Brushe et al., 2024), household noise levels (Simon et al., 2022), background sounds (Suarez-Rivera et al., 2024), music input and episodes (Hippe et al., 2024; Lerma-Arregocés and Pérez-Moreno, 2024; Mendoza and Fausey, 2022) or sibling interactions (Laing and Bergelson, 2024). Recording tools must be capable of encoding these influences while addressing technical challenges such as speaker differentiation and detection of overlapping speech. Beyond the complexity of the environment around the infant, producing speech requires the coordination and activity of multiple biological processes, from the brain to the orofacial articulators, the body, and the autonomic nervous system. Measuring these indices presents unique challenges which multiple open source or non-commercial devices (Maitha et al., 2020, Geangu et al., 2023; Islam et al., 2024), software (Borjon et al., 2024), and algorithms (Xu et al., 2020; Zhang Y. et al., 2024; Mason et al., 2024) have been developed to address. Continued efforts in linking multiple modalities of infant behaviour during language production and perception, particularly in diverse naturalistic settings, will benefit from consortium efforts in agreed-upon construct definitions, synchronisation protocols, and analysis pipelines. Furthermore, methodological advances allow for including multimodal data, such as video, accelerometry, or physiological measures (e.g., Abney et al., 2021; Madden-Rusnak et al., 2024; Wass et al., 2022; Smith et al., 2023; Borjon et al., 2024; Sullivan et al., 2021), alongside audio recordings, could provide rich insights into language environments and developmental trajectories.

## 5. Conclusions

We performed a systematic review of the literature on technology for automated analysis of audio data for studying children’s speech-language experiences. The review identified a large body of studies utilising the LENA system to study language development in a variety of contexts, with alternative free- and/or open-source tools emerging more recently and offering new possibilities for multi-site collaborations. Our review identified gaps in the diversity of cultural, linguistic, geographic, clinical, and social contexts represented in the literature. We also observed limits in the currently available technology, especially on the software end, that, in turn, limit how much researchers can capitalise on their ability to capture real-world audio from children. Achieving global applicability and accessibility requires a holistic approach that integrates technological innovation, methodological rigour, and ethical responsibility. By fostering inclusivity in participant samples, simplifying access to tools, addressing data privacy concerns, and expanding clinical applications, we will move toward a more comprehensive, ecologically valid and equitable understanding of early speech-language development. Capturing the full range of its variability across populations and contexts is key to informing theory-building for typical and atypical language acquisition.
